# Relating to Out-Patients Only

**Published:** 1911-01-28

**Authors:** R. Godwin Chase

**Affiliations:** Sewardsley, Chesterfield.


### Institutional Letter-Box.

### RELATING TO OUT-PATIENTS ONLY.

*To the Editor of* The Hospital.

Sir,—The reason "Provincial" is in the heading is because the scheme relates to those hospitals where members of the honorary staff are general practitioners in practice locally, and not consultants only. By "out-patients" are meant those cases in whom there is some chronic trouble but who are in no sense urgent cases.

It is necessary to approach the question from two points of view: (1) Financial relationship to the Board of Management; (2) financial relationship to the medical men in its district.

(1) Whatever system is adopted for the admission of out-patients to the hospital, it must be one that encourages subscribers to contribute to the expenses of management, drugs, nursing staff, etc. Whether the out-patient letter does this is a debatable point; probably it does, for the subscriber obtains something for his money which, when given to a would-be patient, guarantees to that would-be patient that his cost to the hospital is being defrayed. It, however, fails to guarantee the equivalent in £ s. d. for the gratuitous time and skill given by the hon. staff, which, in the case of an undeserving patient—a case of abuse—some medical man in the district should have legitimate claim to. Of course, in the case of a deserving patient this equivalent becomes nil, because by a deserving case is meant one who is not able to pay a general practitioner's fees.

The alternative to the letter system is to allow anyone to come up to the hospital where he would be interviewed by an expert almoner, and if necessary his home visited. The almoner then decides whether he is a suitable case for gratuitous skill and drugs.

The almoner works on a wage-limit scale. This is always unsatisfactory. From experience it is found that many subscribers do not inquire into the circumstances of would-be patients, although they are supposed to do so. It is quite easy to understand that the small shopkeeper may offend his customers if he does so; hence he forbears.

There would, therefore, seem to be need of some further guarantee from the financial medical point of view. It is suggested that this need would be met by the would-be patient obtaining his own doctor's signature as a countersign to the out-patient letter.

This brings us to paragraph (2), the financial medical point of view. If a doctor, who knows the man, is willing that that man shall have "free medical time and skill," the hon. staff of the hospital can have no cause whatever to complain, for that, among other things, is the nature of the work they have undertaken, and are only too pleased to do. The scheme involves no wages scale. At the very worst it only involves the man telling his own doctor his private difficulties. A man frequently tells his own doctor things more private than that.

The people who will not make this small sacrifice of pride should certainly not be suitable persons for "free medical time and skill." Again, it is a very open, plain, straightforward relationship between the doctor and the would-be hospital patient. These same questions are at the present time successfully dealt with by the doctors, and their patients, in private practice, so why not extend it to the hospital practice too? Finally, from a purely business point of view, the doctors are the ones who suffer financially in the case of abuse. It is therefore only right that they should say who they think are fit people for their brother practitioners to give their time and skill to gratis, while others—i.e. subscribers—guarantee the cost of such treatment as may be ordered.

There arises three difficulties with the scheme: (*a*) Those who have no doctor; (*b*) club patients; (*c*) practitioners guilty of unprofessional conduct.

(*a*) This is not a serious difficulty. It could be left to the discretion of the hospital secretary.

(*b*) This also is not serious. As a precaution against the possible abuse of the hospital by club doctors it could be made the rule that the member obtain his letter from the club. The secretary would then have some criterion that they were being properly used.

(*c*) The signature of these men would not be recognised. Here it is probably right to mention that these men, especially those attached to medical aid societies, are hopelessly overworked; they are therefore only too pleased to send their patients to the hospital where other doctors may do their work for them, and where the letter is paid for by anyone rather than the medical aid society. Again, in these cases they are the paid servants of a lay committee who make rules for their conduct which totally disregard the ethical relationships of one doctor to another. The members of the hon. staff or of the general practitioners in the district will not meet them in consultation. Why, therefore, should the staff help them in their unprofessional conduct by giving gratis their skill and time, even though some subscriber by giving a letter guarantees the cost of the treatment if it be ordered?

The points in favour of the scheme are that it does not upset the subscriber and the subscriptions. It stops medical aid society abuse. There can be no cause of complaint from the honorary staff or from the general practitioner; while undeserving cases will not face their doctors, the deserving will still obtain with a clear conscience their free treatment. It applies only to out-patients, which are cases that are not urgent.—Yours truly,

Sewardsley, Chesterfield. R. Godwin Chase.

